# Breastfeeding moderates the association between family socioeconomic status and child behavior scores

**DOI:** 10.3389/fpubh.2025.1653185

**Published:** 2026-02-03

**Authors:** Sarah E. Turner, Leslie Roos, Nathan C. Nickel, Jacqueline Pei, Sukhpreet K. Tamana, Theo J. Moraes, Stuart E. Turvey, Elinor Simons, Padmaja Subbarao, Piushkumar J. Mandhane, Meghan B. Azad

**Affiliations:** 1 Manitoba Interdisciplinary Lactation Centre (MILC), Winnipeg, MB, Canada; 2 Department of Community Health Sciences, University of Manitoba, Winnipeg, MB, Canada; 3 Manitoba Centre for Health Policy, University of Manitoba, Winnipeg, MB, Canada; 4 Children’s Hospital Research Institute of Manitoba, Winnipeg, MB, Canada; 5 Department of Psychology, University of Manitoba, Winnipeg, MB, Canada; 6 Faculty of Kinesiology and Recreation Management, University of Manitoba, Winnipeg, MB, Canada; 7 School and Clinical Child Psychology Program, University of Alberta, Edmonton, AB, Canada; 8 Department of Pediatrics, University of British Columbia, Vancouver, BC, Canada; 9 Department of Pediatrics, The Hospital for Sick Children, University of Toronto, Toronto, ON, Canada; 10 Department of Pediatrics and Child Health, University of Manitoba, Winnipeg, MB, Canada; 11 Department of Pediatrics, Physiology & Dalla Lana School of Public Health, The Hospital for Sick Children, University of Toronto, Toronto, ON, Canada; 12 Department of Pediatrics, University of Alberta, Edmonton, AB, Canada; 13 Faculty of Medicine and Health Sciences, UCSI University, Kuala Lumpur, Malaysia

**Keywords:** breastfeeding, Child Behavior Checklist (CBCL), family socioeconomic status, cohort, interaction

## Abstract

**Background:**

Children living in low socioeconomic status (SES) environments are more likely to develop behavior problems. Breastfeeding is one behavior that has been positively linked to mental health throughout childhood. We investigated whether breastfeeding modifies the association between low SES and behavior problems.

**Methods:**

We studied a subset of the Canadian CHILD cohort (*N* = 2,342). Lower SES (*n* = 592) was defined as one or more of: (1) low income based on family size, (2) single parenthood, or (3) maternal education below a post-secondary degree. Breastfeeding was reported by caregivers from birth to 2 years. The Child Behavior Checklist (mean 50, SD (10), comprising internalizing, externalizing, and total behavior scores) was administered at 5 years. We tested main effects and interactions between SES and breastfeeding on child behavior, adjusting for several maternal and child characteristics.

**Results:**

Lower SES was related to higher (worse) behavior scores (*B* = 2.06 [95%CI: 1.06, 3.07] for total behavior scores), while longer and more exclusive breastfeeding was related to lower (better) behavior scores (*B* = −2.43 [95% CI: −3.74, −1.11] for exclusive breastfeeding at 6 months, compared to no breastfeeding, for total behavior scores). We observed significant interactions between longer and more exclusive breastfeeding and family SES on internalizing and total behavior scores, indicating that the “socioeconomic gap” in behavior scores becomes smaller with more exclusive and longer breastfeeding.

**Conclusion:**

This study provides new evidence that breastfeeding may be one factor that can help reduce socioeconomic inequities in child behavior scores.

## Introduction

Low socioeconomic status early in life is related to a higher risk of child and adolescent behavior problems (1, 2). Previous research has shown that children living in low-income or single-parent households (1), or having a parent with less than a post-secondary education (3) are more likely to experience mental health challenges, including behavior problems. The reason for these associations is guided by the Family Stress Model, which describes how economic stress can create stress within the family unit, leading to emotional distress, disrupted parenting, and the potential outcomes of child and adolescent behavioral problems (4).

Breastfeeding is one behavior that has been positively linked to improved child behavior (5), partly through the pathways of improved parent–child relationships (5) and infant attachment (6). In alignment with the Family Stress Model, breastfeeding may act as a resource that disrupts the connection between socioeconomic stress and child behavior problems by facilitating positive parent–child relationships and secure attachment between the parent and child. These positive factors may reduce the negative effects of socioeconomic stress. While breastfeeding may be one factor that facilitates this pathway, it is important to note that the development of secure parent–child relationships and attachment is possible and available to families who do not breastfeed through other pathways such as responsive parenting and warm interactions (7, 8).

In high-income countries, families living with low socioeconomic status experience more barriers to breastfeeding, such as inconsistent social support (9), and psychosocial stress (10) and are less likely to breastfeed their children (11). However, previous study has shown that breastfeeding has a stronger association with positive child cognitive outcomes for those who are already experiencing risk factors (12, 13). Whether the moderating role of breastfeeding extends to other outcomes, such as child behavior, is unknown and has not been previously examined. Therefore, our exploratory research aimed to determine if breastfeeding moderates the association between family SES and child behavior scores.

## Methods

### Data and participants

We used data from the CHILD study (www.childstudy.ca), a longitudinal pregnancy cohort of healthy full-term singletons ongoing since 2009 (*N* = 3,296) (14), recruited from four Canadian sites: Toronto, Manitoba, Edmonton, and Vancouver. Children were excluded from the current study if Trisomy 21 was reported (Down Syndrome, *n* = 6), key post-natal data were missing (*n* = 21), or behavior was not assessed (*n* = 927), leaving a total of *N* = 2,342 for analysis. In a previous publication using the same dataset (5), we compared demographic characteristics between those with behavior data (*n =* 2,342) and those with missing behavior data (*n* = 927) and found that children with missing behavior data were more likely to receive no breastmilk and have a mother with lower maternal education, but no differences were observed for birthweight or birth mode.

### Ethics statement

Written informed consent was obtained from all participants at enrollment. This research was approved by Health Research Ethics Boards at the University of Alberta (ID: Pro00118723_REN3), University of British Colombia (ID: H07-03120), University of Manitoba (ID: HS10696 (H2007:255)), SickKids (ID: 1000060059) and McMaster University (ID: 11008).

### Infant feeding, child behavior, and family socioeconomic status

Infant feeding was measured in five ways: any breastfeeding for 1 week or more (yes/no), breastfeeding status at three and six months (none [formula only], partial breastfeeding [breast milk plus formula or other foods] or exclusive breastfeeding), breastfeeding mode at 3 months (where “exclusive breastfeeding” is further divided into “some expressed” or “all direct”), and breastfeeding duration to 24 months. Child behavior was measured at 5 years using the 99-item, parent-reported, Child Behavior Checklist (CBCL) (15, 16), which consists of three scales: internalizing, externalizing, and total behavior problem scores, standardized to a mean of 50 and standard deviation of 10. The mean test–retest reliability of the CBCL scales is high with an *r* of 0.85 (17).

A family SES composite score was derived from three variables that align with a previously developed SES score (18). All variables were measured at 18 weeks of pregnancy: (1) low income based on family size, determined by Statistics Canada low income cutoffs for the year 2012 (19) (the final year of CHILD recruitment); (2) single parent status, if the mother was single, never married, divorced, or separated (as opposed to married or common-law partnership); and (3) low maternal education, defined as less than a college or university degree. Families with one or more SES risk factors were considered “lower SES.”

### Confounders

Nine confounders were considered: child sex, birth weight, gestational age; maternal race (White, Asian [including East, West, and South Asian], or Other), birth mode (vaginal or cesarean), prenatal depression (measured using the Centre for Epidemiological Studies Depression Scale (20) and dichotomized at the clinical cut off of ≥16), older siblings, study site, and attention deficit hyperactivity disorder genetic risk score.

### Statistical analysis

Demographics were compared between those with lower vs. higher family SES, using chi-squared (categorical variables) and Wilcoxon Rank Sum tests (continuous variables). Adjusted linear regression was used to assess the main effects of family SES and breastfeeding on child behavior, followed by a separate set of adjusted interaction models that included an interaction term between family SES and breastfeeding to determine the moderating role of breastfeeding on the SES–behavior relationship. To enhance interpretation of the interactions, we also calculated the statistical significance of the relationship between breastfeeding and CBCL scores for higher and lower SES individually using ANOVA for breastfeeding status at 6 months (categorical variable) and linear regression for breastfeeding duration (continuous variable). Given the modest scope of *a priori* comparisons (three CBCL scores and five breastfeeding measures) in this exploratory research, we did not apply formal multiple-comparison corrections, as these can be overly conservative and obscure clinically relevant associations that can inform future studies. Complete case analysis was employed for all models, and all analyses were done in R version 4.2.1 and RStudio 22.07.1 (21).

## Results

The study population, stratified by higher vs. lower family SES, is described in Table 1. While 72.0% of the sample had no SES risk factors, 19.1% had one risk factor and 8.8% had two or three. As expected, families with lower SES were less likely to breastfeed; 13.9% were exclusively breastfeeding at 6 months with a mean duration of any breastfeeding of 9.9 months (standard deviation [SD] = 7.1), compared to 20.1% of higher SES families exclusively breastfeeding at 6 months with a mean duration of 11.6 months [SD = 6.6; *p* < 0.001 for both breastfeeding variables]. Child behavior scores were also elevated in families with lower SES, with a mean total behavior score of 42.9 [SD = 10.3], compared to a mean total score of 40.6 [SD = 8.5] in the high SES group (*p* < 0.001, however, Cohen’s d to measure the effect size of the difference is small at −0.26 [95% CI: = − 0.36, −0.17]). Families with lower SES were more likely to experience prenatal maternal depression (*p* < 0.001) and have three or more children (*p* < 0.001).

**Table 1:** Characteristics of the CHILD cohort with child behavior data, stratified by family SES (*n* = 2,118).

Variable	*n* (%) or Mean [SD]		
	Higher SES (No SES risk factors; *n* = 1,526)	Lower SES (one or more SES risk factors; *n* = 592)	Chi Square or Wilcoxon Test *p*-value
**Breastfeeding measures**			
Ever breastfed			
No	39 (2.6)	31 (5.3)	0.002
Yes	1,486 (97.4)	558 (94.7)	
Breastfeeding mode 3 months			
Formula only	142 (9.5)	117 (20.8)	<0.001
Breast milk and formula	394 (26.4)	132 (23.4)	
Breast milk only (some expressed)	533 (35.8)	162 (28.8)	
Breast milk only (all direct)	421 (28.3)	152 (27.0)	
Breastfeeding status 3 months			
None	145 (9.5)	119 (20.3)	<0.001
Partial	407 (26.7)	136 (23.2)	
Exclusive	972 (63.8)	331 (56.5)	
Breastfeeding status 6 months			
None	268 (17.8)	173 (30.0)	<0.001
Partial	938 (62.2)	324 (56.2)	
Exclusive	303 (20.1)	80 (13.9)	
Breastfeeding to 24 months (months)	11.6 [6.6]	9.9 [7.1]	<0.001
**Child Behavior Checklist 5 years**			
Internalizing behavior	44.0 [8.7]	45.8 [9.8]	<0.001
Externalizing behavior	39.1 [9.2]	41.3 [10.6]	<0.001
Total behavior	40.6 [8.5]	42.9 [10.3]	<0.001
**Confounders and moderators**			
Child sex			
Female	713 (46.7)	288 (48.6)	0.426
Male	813 (53.3)	304 (51.4)	
Income			
$0–29,999	0 (0.0)	79 (13.4)	<0.001
$30,000–59,999	120 (7.9)	179 (30.3)	
$60,000–99,999	429 (28.1)	154 (26.1)	
$100,000 +	977 (64.0)	120 (20.3)	
Prefer not to say	0 (0.0)	59 (10.0)	
Low income based on family size^a^			
Not low income	1,526 (100.0)	356 (68.6)	<0.001
Low income	0 (0.0)	163 (31.4)	
Maternal education^a^			
Less than high school	0 (0.0)	3 (0.5)	<0.001
Some or completed high school	0 (0.0)	149 (25.2)	
Some college or university	0 (0.0)	323 (54.6)	
Completed college or university	1,526 (100.0)	117 (19.8)	
Maternal marital status^a^			
Married/Common law	1,526 (100.0)	479 (81.7)	<0.001
Divorced/Separated/Never Married/Single	0 (0.0)	107 (18.3)	
Maternal race			
White	1,181 (77.4)	419 (71.0)	<0.001
Asian	234 (15.3)	76 (12.9)	
Other	110 (7.2)	95 (16.1)	
Birth mode			
Vaginal	1,138 (75.7)	442 (75.7)	0.988
C-section	365 (24.3)	142 (24.3)	
Birthweight (kg)	3.5 [0.5]	3.5 [0.5]	0.888
Gestational age (weeks)	39.2 [1.4]	39.2 [1.4]	0.216
Older Siblings			
None	829 (54.3)	297 (50.2)	<0.001
One	543 (35.6)	175 (29.6)	
Two or more	154 (10.1)	120 (20.3)	
Prenatal maternal depression^b^			
Score < 16	1,267 (87.8)	406 (74.8)	<0.001
Score ≥ 16	176 (12.2)	137 (25.2)	
Site			
Edmonton	361 (23.7)	140 (23.6)	<0.001
Toronto	358 (23.5)	88 (14.9)	
Vancouver	424 (27.8)	91 (15.4)	
Manitoba	383 (25.1)	273 (46.1)	
ADHD genetic risk score	−0.01 [1.0]	−0.02 [1.0]	0.774
Family socioeconomic status^c^			
No risk factors	1,526 (100.0)	0 (0.0)	<0.001
One risk factor	0 (0.0)	405 (68.4)	
Two risk factors	0 (0.0)	152 (25.7)	
Three risk factors	0 (0.0)	35 (5.9)	

Chi-squared test of independence is used to test differences between categorical variables. Wilcoxon Rank Sum Test is used to test differences between continuous variables. CBCL = Child Behavior Checklist, ADHD = attention deficit hyperactivity disorder. SD = Standard Deviation. ^a^These variables comprise the family socioeconomic risk variable, ^b^prenatal depression is measured using the Center for Epidemiological Studies Depression Scale (scores ≥ 16 indicate those at risk for clinically significant depression symptoms), ^c^Family socioeconomic risk is comprised of three variables: (1) low income based on family size, (2) mother is single, divorced, widowed or never married, and (3) mother has less than a college or university degree, all measured at 18 weeks of pregnancy. For interaction models, family socioeconomic risk is collapsed into no risk factors (higher SES) vs. one or more (lower SES).

In adjusted regression models, lower family SES was related to higher (worse) total behavior scores (2.06 [95%CI: 1.06, 3.07] point increase, or approximately 1/5 of a standard deviation), while breastfeeding exclusivity and duration were related to lower (better) total behavior scores in a dose-dependent manner (−2.43 [95% CI: −3.74, −1.11] for exclusive breastfeeding, and −1.17 [95%CI: −2.22, −0.11] for partial breastfeeding; −0.07 [95% CI: −0.13, −0.003] for each month of breastfeeding, Table 2). The same direction and similar magnitude of effect were seen for both internalizing and externalizing behavior scores.

**Table 2:** Adjusted main effects and interactions between family SES, breastfeeding, and child behavior at 5 years.

Independent variables		Child Behavior Checklist scales		
		Internalizing	Externalizing	Total
Main effect models		B (95% CI)	B (95% CI)	B (95% CI)
Family SES (*n* = 1,710)	One or more risk factors (lower SES) (reference: no risk factors [higher SES])	1.53 (0.54, 2.53)**	**1.88** (0.81, 2.95)***	**2.06** (1.06, 3.07)***
Breastfeeding at 6 months (*n* = 1,851)	No Breastfeeding (formula only)	reference	reference	reference
	Partial breastfeeding	**−1.19** (−2.24, −0.13)*	−0.77 (−1.89, 0.35)	**−1.17** (−2.22, −0.11)*
	Exclusive breastfeeding	**−2.42** (−3.74, −1.10)***	**−2.04** (−3.44, −0.64)**	**−2.43** (−3.74, −1.11)***
Breastfeeding duration (*n* = 1,873)	Breastfeeding duration to 24 months (per month)	**−0.09** (−0.15, −0.03)**	−0.04 (−0.11, 0.03)	**−0.07** (−0.13, −0.003)*

Interaction models between family SES and breastfeeding		Interaction *p*-value	Interaction *p*-value	Interaction *p*-value
Family SES X Breastfeeding at 6 months (*n* = 1,687)	No Breastfeeding (formula only)	reference	reference	reference
	Partial breastfeeding	0.25	0.59	0.28
	Exclusive breastfeeding	**0.01** (see Figure 1A)	0.06 (see Figure 1C)	**0.01** (see Figure 1E)
Family SES X Breastfeeding duration (*n* = 1,708)	Breastfeeding duration to 24 months (per month)	**0.03** (see Figure 1B)	0.11 (see Figure 1D)	**0.02** (see Figure 1F)

**Figure 1:**
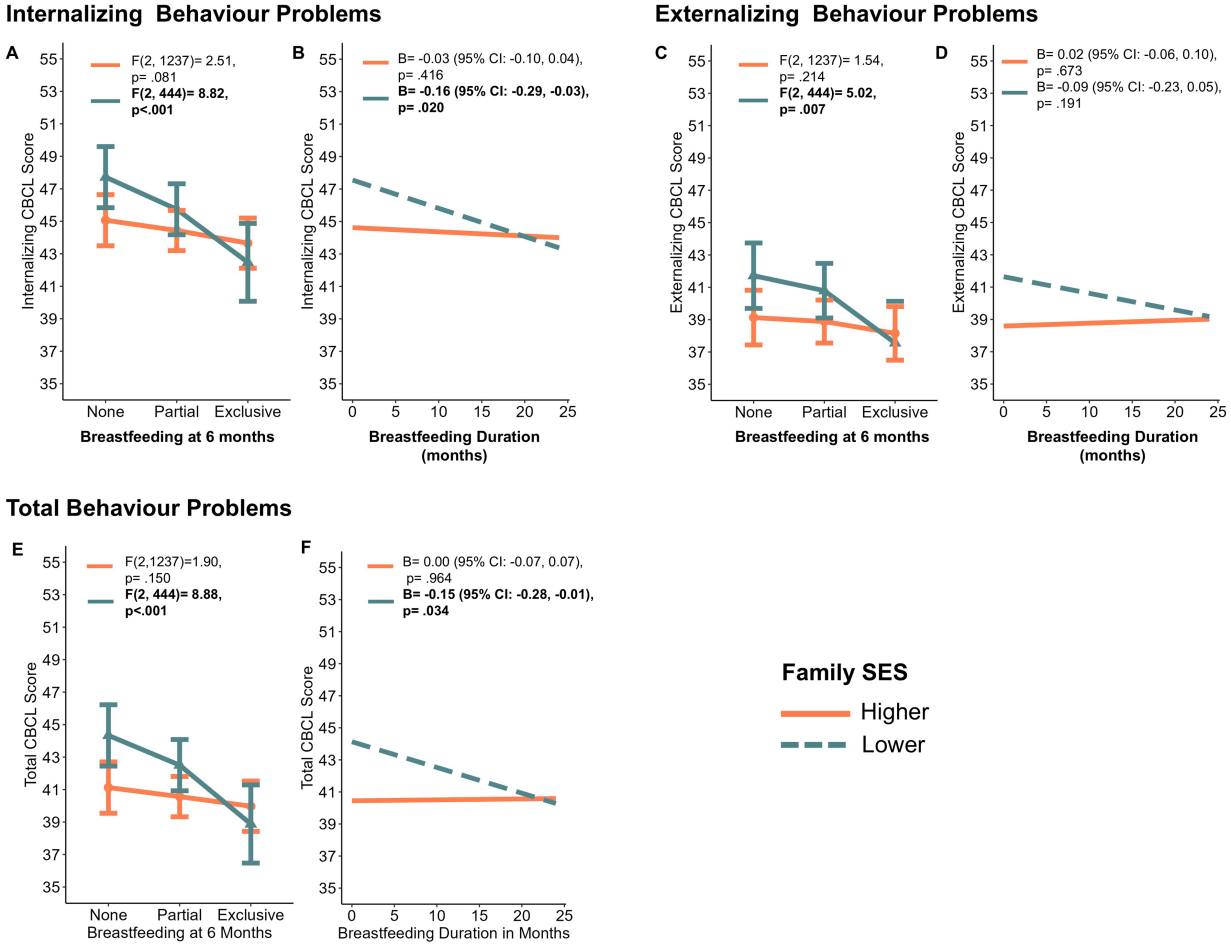
Interaction plots depicting interactions between breastfeeding and family socioeconomic risk on child behavior checklist scores. A, C, E. show the interaction between breastfeeding status at 6 months and family SES on internalizing, externalizing and total behavior problems, respectively. B, D, F show the interaction between breastfeeding duration and family SES on internalizing, externalizing and total behavior problems, respectively. CBCL, Child Behavior Checklist (lower scores indicate better behavior); SES, socioeconomic status; ANOVA, analysis of variance. Plots are based on interactions shown in Table 2. ANOVA F-test and linear regression B values show the difference between means (ANOVA) or the slope (regression) between breastfeeding and CBCL scores for lower and higher SES groups.

Main effects models report associations between family SES and CBCL scores and breastfeeding and CBCL scores, tested in nine separate regression models. Six separate interaction models report interaction *p*-values from models that include an interaction term between family SES and breastfeeding for each CBCL outcome. Complete regression model statistics for all main effects and interaction models can be found in Supplementary Table 1. Interactions are graphically depicted in Figure 1. All models are adjusted for: child sex, maternal race, birth mode, birthweight, gestational age, older siblings, prenatal maternal depression, study site, and attention deficit hyperactivity disorder genetic risk score. Lower family SES is comprised of having one or more of the following: (1) low income based on family size, determined by Statistics Canada low income cutoffs for the year 2012, (2) mother is single, divorced, widowed, or never married, (3) mother has less than a post-secondary education, all measured at 18 weeks of pregnancy. CBCL, Child Behavior Checklist (lower scores indicate better behavior); SES, socioeconomic status. Values in bold indicate significant effects (*p* ≤ 0.05). * *p* ≤ 0.05, ** *p* ≤ 0.01, ***p < 0.001.

Significant adjusted interactions were observed between family SES, and exclusive breastfeeding at 6 months, for both internalizing and total behavior scores (*p* < 0.05 for internalizing scores and *p* < 0.01 for total scores). A marginally significant interaction was observed between family SES and exclusive breastfeeding at 6 months for externalizing behavior scores (*p* = 0.06). Significant adjusted interactions were observed between family SES and breastfeeding duration for both internalizing and total behavior scores (*p* < 0.05 for both internalizing and total scores), although this interaction was not significant for externalizing behavior scores (*p* = 0.11). Complete regression model statistics, including interaction beta coefficients and partial eta-squared effect sizes, can be found in Supplementary Table 1. To enhance interpretation of the interactions, Figures 1A–F show the change in internalizing, externalizing, and total CBCL scores by breastfeeding for both lower and higher family SES groups. These interaction plots show that the “socioeconomic gap” in behavior scores becomes smaller with more exclusive and longer breastfeeding. For example, among those with higher SES, there is little change in total behavior scores with more exclusive breastfeeding (i.e., no significant differences between breastfeeding levels, calculated using ANOVA, *F*(2, 1,237) = 1.90, *p* = 0.150, Figure 1E, orange line), while for those with lower SES, more exclusive breastfeeding is related to better total behavior scores (calculated using ANOVA, *F*(2, 444) = 8.88, *p*<0.001, Figure 1E, green line). The direction of effects and interpretation is the same for panels A, B, C, and F. No interactions were observed for other breastfeeding exposures (i.e., ever breastfeeding, breastfeeding mode, or breastfeeding exclusivity at 3 months).

## Discussion

This exploratory Canadian study shows that the association between family SES and child internalizing and total behavior scores is moderated by breastfeeding exclusivity and duration, suggesting that breastfeeding may be one factor that can help “close the gap” in behavior problems experienced disproportionately by children living with lower SES. These associations remained after adjustment for important confounders including prenatal maternal depression and number of older siblings.

Notably, we did not observe statistically significant interactions with externalizing behavior problems; however, the direction and magnitude of the main effects, interaction coefficients, and visualization of change in externalizing scores by breastfeeding for lower and higher SES groups were similar to internalizing and total behavior problems. Previous studies have shown that the link between breastfeeding and internalizing behavior is partially due to improved mother–child relationships (5, 22), which is one factor that may disrupt the connection between socioeconomic stress and child behavior problems. This mechanism may not apply as strongly to the outcome of externalizing behavior which would explain the lack of statistically significant findings here.

While the CBCL effect sizes in the main effects and interaction models are small (all less than a one SD or 10-point change) and may not represent individual clinical relevance, small effect sizes can still have population-level relevance. This is explained by Roses Theorem which states that small effect sizes can be meaningful at the population-level because a large number of people at a small risk can result in more incidence of the outcome than a small number of people at a high risk (23).

Similar to our findings suggesting differential breastfeeding effects depending on SES risk, another study in the United States showed that breastfeeding exclusivity and duration were related to lower prevalence of conduct problems in kindergarten for those with high genetic risk, with a much smaller association for those with low genetic risk (13). These findings suggest that those in high-risk environments (i.e., socioeconomic or genetic) will benefit more from health-promoting early life exposures (such as breastfeeding) than those in lower risk environments.

Our results are particularly important because those with lower SES are typically the populations that experience more barriers to breastfeeding and have lower breastfeeding rates (observed in other studies (24) and confirmed here in the CHILD cohort). The reasons for this are multifactorial and span the social determinants of health. For example, families living with lower SES are more likely to experience food insecurity, unstable housing or homelessness, inconsistent employment, mental health concerns, and less consistent social support, all of which add extra family stress and can impact breast milk production, the ability to access breastfeeding support, or limit the time available to breastfeed (9, 25, 26). For breastfeeding to be a possible choice for all families, policies and programs that reduce barriers to breastfeeding among those living in low SES environments are necessary (27). The results of this study support programs like the Canadian Prenatal Nutrition Program that provide targeted support to those living in more disadvantaged settings to help close the health equity gap between those living in advantaged settings (28). This study also supports policies and programs to increase SES, such as basic annual income and increasing the Canada Child Benefit to promote equity and reduce child poverty (29). Based on the current findings, breastfeeding may be one strategy that could help narrow the gap in child behavior scores between those with lower vs. higher SES.

## Limitations

The CHILD cohort comprises relatively higher SES families with fewer child behavior problems compared to the general Canadian population and the CBCL normative population (11, 17). Therefore, our results may underrepresent the true associations of family SES and breastfeeding with child behavior. Furthermore, the family SES variable has not been validated; the use of a composite score to define SES has been used previously, but with low education defined as less than a high school degree, instead of less than college/university which we used here due to very few mothers with less than a high school degree in our sample (18). Thirdly, unmeasured residual confounding from variables such as parenting practices, early child temperament, and infant health problems may account for some or all of the observed associations. Finally, this study was underpowered to examine nuanced interactions with a three-level SES variable, requiring further categorization which limited the resolution of this analysis.

## Conclusion

This study provides new evidence that breastfeeding may be one factor that can help “close the SES gap” in child behavior scores. This evidence highlights the importance of breastfeeding support services, particularly in populations with lower SES.

## Data Availability

The data analyzed in this study is subject to the following licenses/restrictions: a list of variables available in the CHILD Cohort Study is available at https://childstudy.ca/for-researchers/study-data/. Researchers interested in collaborating on a project and accessing CHILD Cohort Study data should contact the Study’s National Coordinating Centre (NCC) to discuss their needs before initiating a formal request. More information about data access for the CHILD Cohort Study can be found at https://childstudy.ca/for-researchers/data-access/. Requests to access these datasets should be directed to child@mcmaster.ca.
